# Lysis with Saponin improves detection of the response through CD203c and CD63 in the basophil activation test after crosslinking of the high affinity IgE receptor FcεRI

**DOI:** 10.1186/1476-7961-3-10

**Published:** 2005-07-04

**Authors:** Hans Jürgen Hoffmann, Mette Bøgebjerg, Lars Peter Nielsen, Ronald Dahl

**Affiliations:** 1 Department of Pulmonary Medicine, Aarhus University Hospital, Aarhus University, DK 8000 Aarhus C, Denmark; 2 Institute of Pharmacology, Aarhus University, DK 8000 Aarhus C, Denmark

## Abstract

**Background:**

The basophil activation test (BAT), in which translocation of markers to the surface of blood basophils is measured in response to allergen by flow cytometry, is a rapid assay that is gaining popularity. Two markers are currently being evaluated for the BAT; CD63 and the lineage-specific CD203c. In a recent report, detection of CD203c after lysis with Saponin was shown to be superior to detection of CD63 after lysis with formic acid. We wanted to compare a) lysis with formic acid and lysis with Saponin, b) the response through CD203c and CD63, and c) the definition 10% activated cells above background with the probability binning metric T(χ) > 4, on sets of data generated with blood basophils stimulated with varying concentrations of anti-FcεRI antibody.

**Methods:**

Blood from volunteers was incubated with serial logarithmic dilutions of anti-FcεRI and subsequently with antibodies to CD203c PE and CD63 FITC. Sets of samples set up in parallel were lysed with either Saponin based Whole Blood Lysing reagent or with formic acid based Immunoprep/Q-prep. Samples were acquired on a FACS Calibur, but were compensated and analysed offline. Responders were defined as persons who had 10% or more activated basophils above background, or a T(χ) > 4, for two consecutive dilutions of anti-FcεRI antibody.

**Results:**

More basophils (median 1164 vs. median 397) and better discrimination of upregulated CD203c and CD63 amongst responders were obtained after lysis with Saponin than after lysis with formic acid. We suggest that CD203c may be a more sensitive marker for the BAT than CD63, as 6/11 responders were found with CD203c, compared with 3/11 with CD63. Most responders (7/11) were identified with probability binning.

**Conclusion:**

A combination of lysis with Saponin and the markers CD203c and CD63 computed by probability binning may be the most sensitive method of detecting activation of basophils after stimulation through FcεRI.

## Background

The basophil activation test (BAT), in which an allergen-specific response is measured by flow cytometry (reviewed in Ebo et al [1]), is gaining popularity as an *ex vivo *diagnostic tool. It is a rapid test with relatively high sensitivity and specificity that relies on surface translocation of transmembrane markers by regulated exocytosis in response to a stimulus through the high affinity IgE receptor (FcεRI). Crosslinking by anti-IgE of IgE bound to FcεRI [2,3], or stimulation with fMLP [4] serve as positive control. A third option is to crosslink FcεRI with a monoclonal antibody [5]. Concentrations of allergens selected to elicit a graded response are used to test for response to allergen. We regard the BAT as an attractive tool in the arsenal of the allergologist to identify culprit allergens.

Two markers are currently evaluated for the BAT – CD63 with a broad expression profile [6] and more recently CD203c, a lineage marker for CD34+ progenitor cells, mast cells and basophil granulocytes [7]. As CD203c is a unique marker for basophils and mast cell precursors, it may be sufficient for identification and detection of activation of basophils. When using CD63 as a metric, it is common to use antibodies to IgE [2,8-10], sometimes with CD45 [11,12] to identify basophils. An alternative method uses CD123 and HLA DR [13].

Most reports on the test have used either one of the markers, but in a recent publication [14] these markers were directly compared – with the caveat that response through CD63 was evaluated after lysis with Q-prep (based on formic acid), and the response through CD203c was evaluated after lysis with Whole Blood Lysing reagent (WBL, based on Saponin), both from Coulter. Although Hauswirth et al [7] have shown that there is good concordance between CD63 and CD203c, authors that established their experience base with CD63 contested the publication of data suggesting that CD203c is superior to CD63 [5,15]. We have compared the two markers CD63 and CD203c after lysis with WBL or Immunoprep/Q-prep, the manual kit from Coulter using the same chemistry as Q-prep, and find that lysis with the Saponin-based WBL is superior to lysis with Immunoprep/Q-prep, and that the response through CD203c after lysis with Saponin is stronger and more distinct than that through CD63. We have also tested probability binning condition T(χ) > 4 as an algorithm to identify a response, and found it comparable to "baseline + 10% activated cells", the method we used to define positive events [14].

## Methods

### Stimulation and flow cytometry

The method used was designed to be rapid for use in routine diagnosis. Heparinised blood (4 ml) was obtained from 11 informed volunteers, of which 2 had allergic airway disease. The procedure had been approved by the Ethics Committee of Aarhus County. Aliquots (100 μl) were incubated at 37°C for 5 minutes with increasing amounts of antibody to FcεRI CRA1 (Kyoto Pharmaceutical Industry Co., Japan) [16](spanning 7 orders of magnitude from 0,01 pg/μl to 10 ng/μh). CD203c PE (Immunotech, France) and CD63 FITC (Caltag, USA) were added to the same tube (titrated to 5 μl for each antibody) and incubation at 37°C continued for 10 minutes. The time point at 15 minutes was selected on the basis of published optimal times of response for CD203c [7,17] and CD63 [17]. The reaction was stopped by addition of lysing reagent, and after lysis, fixation and a wash, the samples were analysed on a FACS Calibur (Becton-Dickinson, Irvine, CA, USA) without hardware compensation. Samples were lysed with either WBL or Immunoprep/Q-prep, (both from Coulter Corporation, Hialeah, FL, USA) according to the manufacturer's instructions. Standards for software compensation were acquired by labelling one drop of Comp beads (Becton-Dickinson, Irvine, CA, USA) with 5 μl of antibody.

### Data analysis and statistics

**Figure 1:**
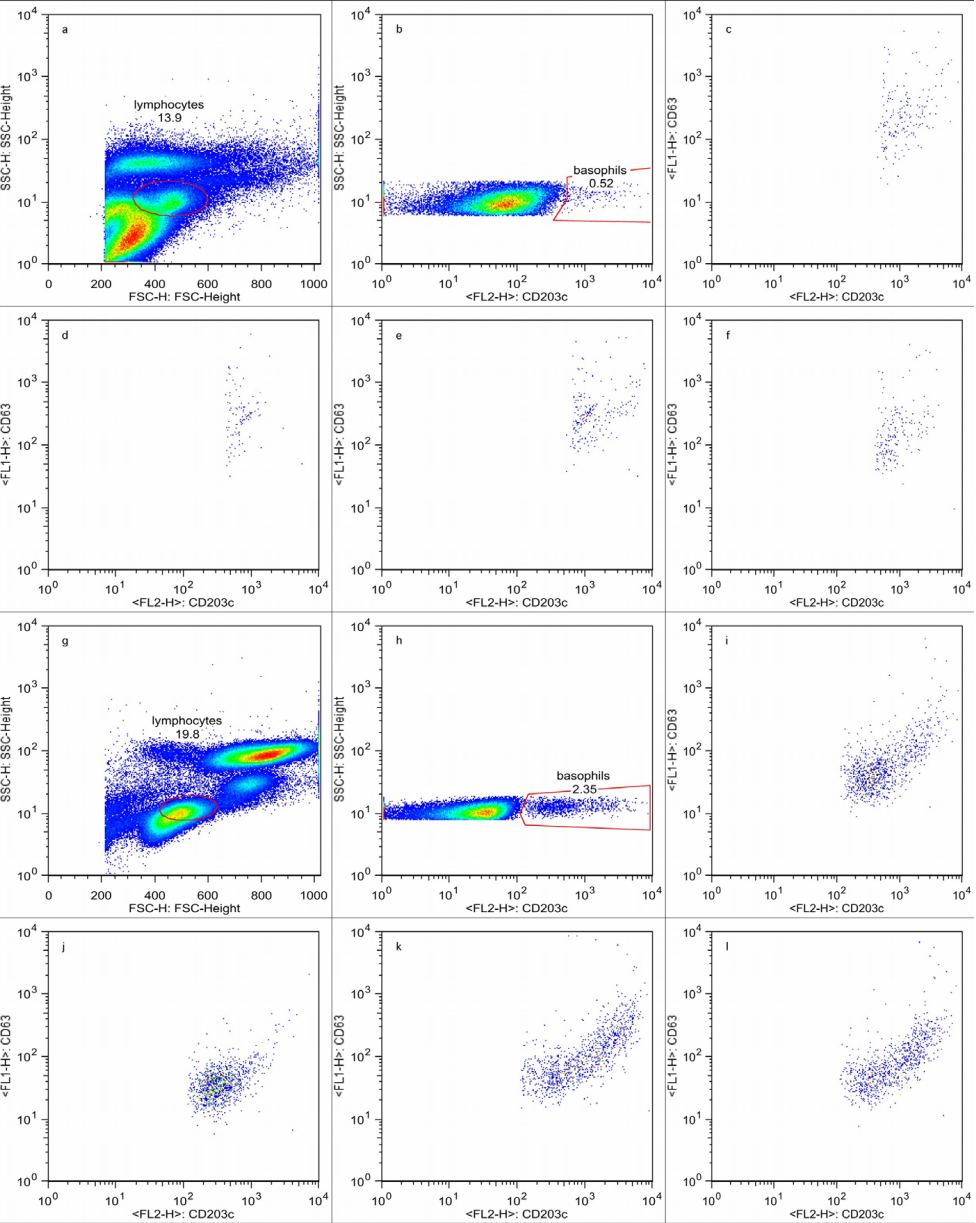
**Analysis of Basophil activation**. Analysis of basophils after lysis by Immunoprep/Q-prep (a–f) or WBL (g–l) of a representative donor. By dynamic backgating (illustrated in figure 2), the region in a forward scatter vs side scatter plot in which basophils are located was optimised (a & g). CD203c+ cells were identified in this gate (b & h), and the expression of CD203c and CD63 was evaluated (c & i). CD203c vs CD63 expression at differend concentrations of CRA1 is shown after both lysis conditions (1 ng/ul panels c, g, 0,001 ng/ul in panels f & l, 0, 0,0001 in panels e & k; pbs in panels d & j).

**Figure 2:**
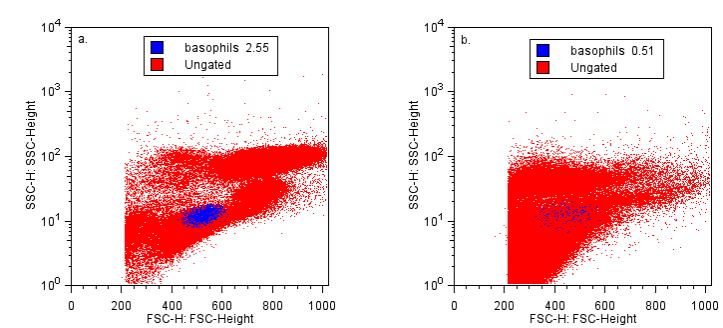
**Backgated basophil populations**. Backgated basophils identified after lysis with Immunoprep/Q-prep (a) and WBL (b).

**Figure 3:**
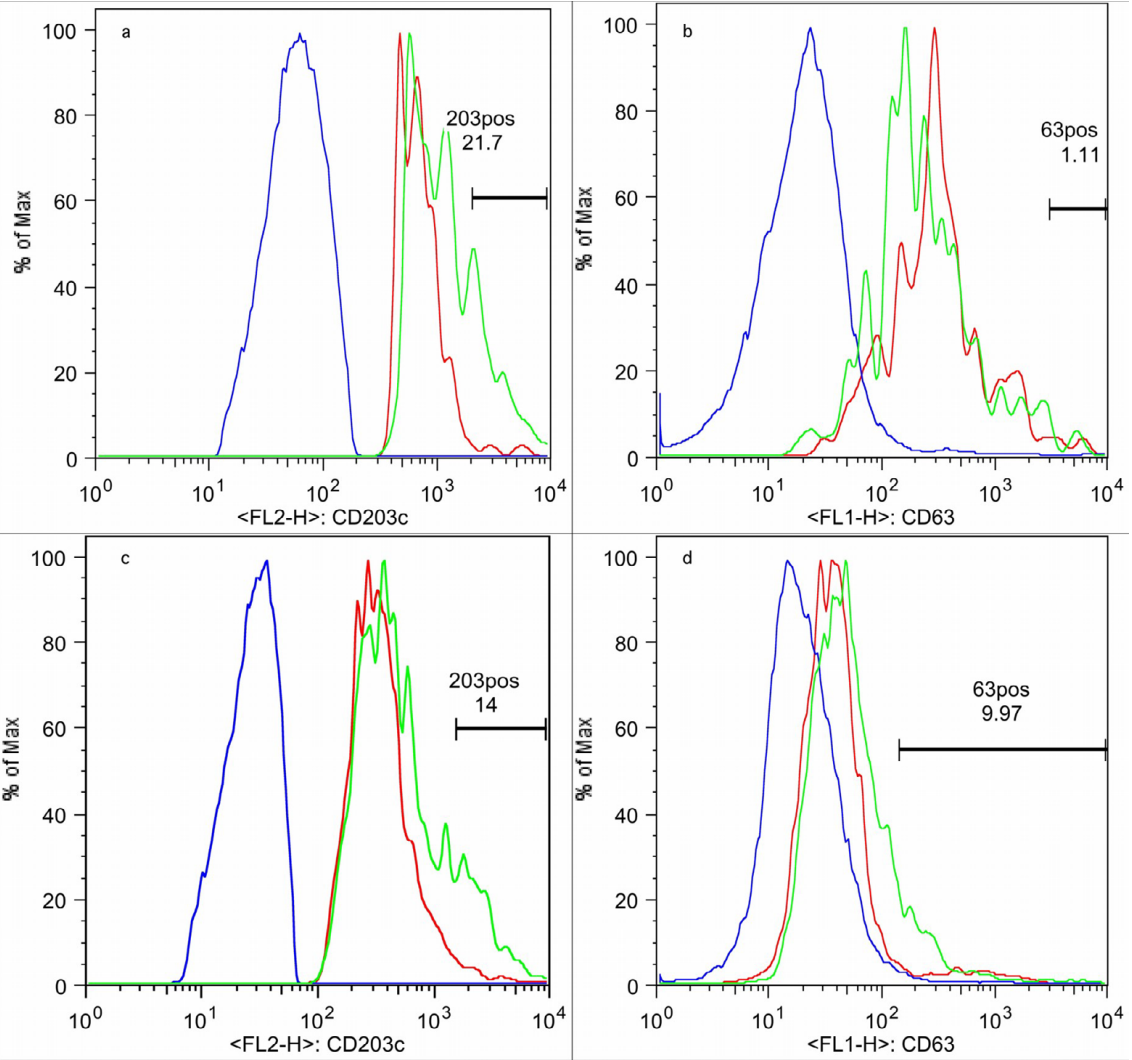
**Histograms of CD63 and CD203c expression on basophils and control cells**. Histograms of expression of CD203c (a & c) and CD63 (b & d) on basophils at baseline (red line) and after maximal stimulation (green line), and on lymphocytes (blue line) after lysis with Immunoprep/Q-prep (a & b) and WBL (c & d). Markers were set to include ~2% of unstimulated basophils.

Data files were compensated and analysed with FlowJo version 6.1 (Treestar Corp, USA, Figure 1). The lymphocyte region containing CD203c^+ ^basophils was confirmed by the dynamic backgating function of FlowJo (Figure 2), and basophils were identified as CD203c^+ ^cells. In a separate dot plot, basophil expression of CD63 and CD203c were plotted. Thresholds were set at 2% on histograms of CD203c and CD63 (Figure 3). Figures 1, 2, 3 were generated on the same representative dataset. For probability binning analysis [18] of cells in the basophil gate, unstimulated samples were set as reference, and all samples stimulated with CRA1 were compared to that sample. Normal distribution of the data sets (% positive cells) was confirmed (SPSS v 10), and data was analysed with the Students t test. P < 0,05 was assumed to be significant.

## Results

### More basophils are detected with WBL than with Immunoprep/Q-prep

The yield of basophils from the WBL lysis (median 1164 cells for 250 000 events acquired) was significantly better than the yield with Immunoprep/Q-prep (median 397 cells for 250 000 events acquired) for 7/11 data sets (Table 1). In the four sets where the difference was not significant, the yield of basophils in the WBL assay was lower than the median, but still better than with Immunoprep/Q-prep. When plotting the cell number against the amount of CRA1 added, there was a trend toward an increased yield at high concentrations of CRA1 after lysis with Immunoprep/Q-prep, suggesting that basophils were detected more easily when they express high levels of CD203c. This trend was much less pronounced after lysis with WBL.

**Table 1:** Basophil cell yields. Average of numbers detected after lysis at 7 different concentrations of CRA1 from 250000 (normalized) events after lysis with either WBL or Immunoprep/Q-prep (average ± SD, tested with a paired t test). + = atopics, - = non atopics

Donor	Allergy	WBL ± SD	Immunoprep ± SD	p-value
1	-	1714 ± 314	1108 ± 449	<0,015
2	-	1979 ± 132	369 ± 274	<0,001
3	+	1638 ± 66	406 ± 262	<0,001
4	-	568 ± 84	182 ± 107	<0.001
5	-	1646 ± 282	371 ± 248	<0,001
6	+	483 ± 113	436 ± 138	0,494
7	-	1164 ± 70	213 ± 160	<0.001
8	-	1132 ± 372	582 ± 150	0,003
9	-	1397 ± 207	306 ± 133	<0,001
10	-	995 ± 481	577 ± 415	0,107
11	-	407 ± 128	397 ± 183	0,904
Median		1164	397	

### CD203c is more sensitive than CD63 at detecting signalling through FcεRI

When defining a positive response as two consecutive responses of more than 10% above baseline [14], fewer responders were recorded with CD63 (3/11 data sets) than with CD203c (6/11 data sets). All responders through CD63 respondent also through CD203c. Lysis procedure had no effect on CD63, but there was one more response detected with CD203c after lysis with WBL than after lysis with Immunoprep/Q-prep (Table 2). The participants were split into three groups on the basis of >10% positive cells at two consecutive dilution (Table 2): responders with both CD63 and CD203c (n = 3), responder with CD203c only (4 for WBL, n = 3 for Immunoprep/Q-prep) and non responders (n = 5).

**Table 2:** Responders as defined by Boumiza et al [14] or by T(χ) > 4 for two consecutive dilutions of CRA1. Y = responder

	CD63		CD203c		T(χ)_63,203c_	
Donor	WBL	Immunoprep	WBL	Immunoprep	WBL	Immunoprep

1						
2						
3						
4					Y	
5						
6			Y		Y	Y
7			Y	Y	Y	Y
8			Y	Y	Y	
9	Y	Y	Y	Y	Y	
10	Y	Y	Y	Y	Y	Y
11	Y	Y	Y	Y	Y	Y

### Probability binning offers an integrative alternative to using either only CD203c or only CD63

When analyzing the same dataset by defining that the probability binning metric T(χ)_CD203c, CD63 _> 4 for two consecutive dilutions as a response, a similar classification emerged for the data set obtained after lysis with WBL (7/11 data sets), and to some part with Immunoprep/Q-prep (4/11 data sets, Table 2). Discrimination of T(χ)_CD203c, CD63 _was significantly better after lysis with WBL than after lysis with Immunoprep/Q-prep (4/11 data sets).

### The ratio of activation was higher for CD203c than for CD63

**Figure 4:**
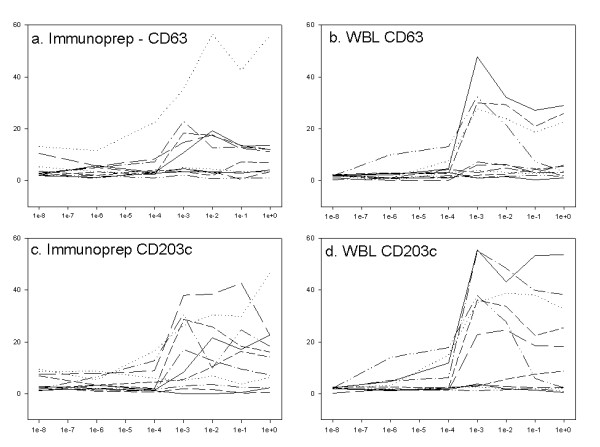
**Comparison of response of CD63 and CD203c with different lysis methods**. Degree of activation at varying concentrations of CRA1. The % positive cells at a given concentration are plotted against the amount of anti-FceRI antibody. The lowest data point is labelled 1e-8 for the purpose of representation on a log scale. (a) Immunoprep/Q-prep lysis, detection with CD63, (b) Immunoprep/Q-prep lysis, detection with CD203c, (c) WBL lysis, detection with CD63, (d) WBL lysis, detection with CD203c. In panel d, one responder achieved significantly higher activation ratios than 35.

The degree of activation detected through CD203c and CD63 after lysis with either Immunoprep/Q-prep or WBL was compared by dividing the fraction of positive cells in CRA1-activated samples of responders by the fraction of positive cell at baseline (Figure 4). The signal was better with WBL (Figure 4b & 4d) than with Immunoprep/Q-prep (Figure 4a & 4c) and was slightly better with CD203c (Figure 4c & 4d) than with CD63 (Figure 4a & 4b). Lysis with WBL was significantly better for both CD203c (3/6 data sets) and for CD63 (1/6 data sets) (Table 3). Detection with CD203c was significantly better after lysis with WBL (1/6 data sets) and Immunoprep/Q-prep (2/6 data sets). Detection of CD203c was significantly better in 4/6 experiments when comparing the lysis condition used by Boumiza et al (Immunoprep/Q-prep for detecting CD63 and WBL for detection of CD203c) [14].

**Table 3:** Difference in activation for combinations of WBL, Immunoprep/Q-prep and CD63 and CD203c amongst responders The p-value was calculated with the Students t-test. ns = not significant

	WBL vs Immunoprep		CD203c vs CD63		
	CD63	CD203c	Immunoprep	WBL,	CD203c WBL vs CD63 Immunoprep

6	ns	0,025	ns	ns	ns
7	ns	0,016	0,027	0,016	0,012
8	0,016	ns	0,004	ns	0,019
9	ns	ns	ns	ns	ns
10	ns	0,018	ns	ns	0,019
11	ns	ns	ns	ns	0,043

## Discussion

The BAT is an exiting development in applied functional flow cytometry, and a number of laboratories have developed independent procedures to use it The first common approach to standardization is a EAACI working paper . We chose to use stimulation of blood basophils through FcεRI with the antibody CRA1 [16] to compare different lysis methods. Recently, Boumiza published a controversial comparison of responses through CD63 after lysis with Immunoprep/Q-prep and CD203c after lysis with WBL [14] that was contested by groups with experience in detecting CD63. Other reports that so far have compared CD63 and CD203c [7,17] give anecdotal evidence of a similar response through the markers, but have not compared them stringently. We had noticed that lysis with Saponin (on which WBL is based) gives appreciably better results than lysis with ammonium chloride (unpublished), and chose to compare the lysis methods (WBL, with Saponin, and Immunoprep/Q-prep lysing reagent, with formic acid) and markers (CD63 FITC and CD203c PE) used for the report)[14].

The yield was remarkably better for WBL (involving Saponin) than for Immunoprep/Q-prep (involving formic acid). Although the minimum number of basophils for a useful test has been reported to be 100 [19], we prefer to have more than 500, which is within reason as we could obtain approximately 500 basophils from 100 μl blood from most donors using lysis with WBL (Table 1).

The threshold for a positive response has in the past been set by an empirically determined fraction of basophils detected above background. The threshold for detection of allergens by a BAT should be set using Receiver Operating Curves [20]. For the present study it was deemed to be stringent to set it to be 10% above the unstimulated control experiment in two consecutive dilutions of allergen or, in this case, antibody to FcεRI to be comparable to the previous study comparing CD63 and CD203c [14]. Using this threshold, 6 of 11 persons mounted a positive response to cross linking of FcεRI. In other studies, the threshold is set empirically between 6% and 17% (EAACI Position paper at ).

As there is evidence that CD203c and CD63 are translocated to the basophil cell surface by different mechanisms and with different kinetics [17], it may be of interest to monitor them simultaneously. Probability binning [21] is an algorithm by which variance in the control distribution is minimised by varying bin size before a normalised chi-square value is computed for each sample distribution using the same bins. This has two advantages: 1. it combines the information residing in cell number in a given bin with median fluorescence intensity and 2. the bins constructed during the analysis can be extended from a univariate analysis to be rectangles on a dot plot of CD203c vs CD63 containing an even number of events of the unstimulated control, and a single chi-square value can then be computed that incorporates change in both markers [18]. We show that the result of probability binning with CD63 and CD203c as dimensions is similar to the result of the method of assigning a threshold at baseline + 10%. This suggests that T(χ)_CD203c CD63 _is as sensitive as the conditions defined by Boumiza et al [14] (background + 10% positive).

The relative sensitivity of CD203c and CD63 was compared by calculating the relative increase in ratio of positive cells over baseline conditions at each concentration of FcεRI antibody. As lysis with Immunoprep/Q-prep results in a lower basophil yield and immunofluorescence than WBL, and CD63 appears to be upregulated to a lesser extent than CD203c, the combination used in [14], detecting CD63 after lysis with Immunoprep/Q-prep (Figure 4a) and detection of CD203c (Figure 4d) after lysis with WBL, accentuated differences between the markers.

## Conclusion

We present data that supports the claim that WBL is a better lysis method than the automated Immunoprep/Q-prep (shown here), and that CD203c is more sensitive than CD63 at detecting FcεRI-mediated activation and uniquely identifies basophils in human blood. As the presented data were obtained with an antibody to FcεRI, the results need to be confirmed after stimulation with allergen. Probability binning offers an approach that combines CD63 and CD203c into one metric that has a high response. A combination of lysis with Saponin and the markers CD203c and CD63 [17] computed by probability binning may be the most sensitive method of detecting activation of basophils after stimulation through FcεRI.

## Competing interests

The author(s) declare that they have no competing interests.

## Authors' contributions

HJH conceived the project, analysed the data and wrote the manuscript. BMH recruited patients and did the experiments, LPN contributed to project design and writing of the manuscript. RD contributed to the design of the study and writing of the manuscript. All authors read and approved the final manuscript.
